# Biocompatible branched copolymer nanoparticles prepared by RAFT polymerization as MRI/PET bimodal tracers

**DOI:** 10.1186/2197-7364-2-S1-A90

**Published:** 2015-05-18

**Authors:** Chang-Tong Yang, He Tao, Alexander W Jackson, Prashant Chandrasekharan, Parasuraman Padmanabhan, Balázs Gulyás, Christer Halldin

**Affiliations:** Lee Kong Chian School of Medicine, Nanyang Technological University, Singapore; Institute of Chemical and Engineering Sciences, Agency for Science Technology and Research, Singapore; Laboratory of Molecular Imaging, Singapore Bioimaging Consortium, Agency for Science Technology and Research, Singapore; Karolinska Institutet, Department of Clinical Neuroscience, Stockholm, Sweden

Stable branched copolymer nanoparticles of varying size (Dh = 20 – 35 nm) have been developed and employed as MRI nano-sized contrast agents. RAFT polymerization has been employed to prepare these novel nanoparticles possessing DO3A macrocycles within their cores and succinimidyl ester benzoate functionalities within their coronas. It has been demonstrated that these nanoparticles can chelate gadolinium and in vitro cytotoxicity studies using HK-2 cells established their negligible toxicity profile. In vivo MRI experiments showed that these nanoparticles have a high relaxivity and a long blood retention time. Xenograft experiments further illustrated the ability of these nanoparticles to perfuse and passively accumulate in tumor cells, presumably through the enhanced EPR effect. The presence of the succinimidyl ester benzoate functionalities within the nanoparticle coronas will permit future surface modification with fluorophores or targeting moieties to generate nanoparticles to study opportunities for bimodal imaging nano-probes or active cell targeting contrast agents. The chelation with PET radioisotopes (68Ga(III) or 64Cu(II)) can afford various PET tracers.

